# Bouncing forward of young refugees: a perspective on resilience research directions

**DOI:** 10.3402/ejpt.v4i0.20124

**Published:** 2013-05-02

**Authors:** Marieke Sleijpen, F. Jackie June ter Heide, Trudy Mooren, Hennie R. Boeije, Rolf J. Kleber

**Affiliations:** 1 Foundation ARQ, Diemen, the Netherlands; 2 Department of Clinical & Health Psychology, Utrecht University, Utrecht, the Netherlands; 3 Foundation Centrum'45, Diemen, the Netherlands; 4 Department of Methodology and Statistics, Utrecht University, Utrecht, the Netherlands

**Keywords:** refugees, youth, trauma, resilience, mixed methods research

## Abstract

While studies on the consequences of trauma and forced migration on young refugees have focused mainly on their pathology, a focus on resilience in young refugees is needed to adequately represent their response to adversity and to help understand their needs. The aim of this article is to present a proposed study of resilience in young refugees which has been informed by an overview of achievements and challenges in the field of resilience.

In order to advance the field of resilience, several topics need clarification: definition and assessment of resilience, the relation of resilience to other constructs and the underlying biological and external factors influencing resilience. With respect to young refugees, the cross-cultural applicability of resilience has to be examined. Qualitative research, mixed method designs, comparative studies, and longitudinal studies seem especially promising in furthering this goal.

The proposed study compares refugee adolescents with Dutch adolescents. Data from qualitative evidence synthesis, interviews, questionnaires, experiments, and DNA analysis will be combined to provide a multifaceted picture of factors contributing to resilience, resulting in a better understanding and efficient use of “resilience” to meet the needs of traumatised youth.

War and persecution around the world force children and adolescents to leave their own country. In 2011, more than 876,000 people worldwide appealed for refugee status, 34% of whom were younger than 18 years (United Nations High Commissioner for Refugees, 2012). Many of these young refugees will grow up to be a part of Western society, shaping its future. Young refugees need to rapidly adapt to changing societal conditions. After their flight, besides having to deal with an often traumatic history, they encounter complex legal immigration processes as well as social, cultural, and linguistic differences between their region of origin and their new setting (e.g., Fazel, Reed, Panter-Brick, & Stein, 2012). It is important to not only understand the consequences of the ordeals that young refugees are faced with but also to examine the factors that are related to resilience and growth in the face of adversity.

Recent systematic reviews have shown that young refugees are at serious risk of developing a range of health and development-related problems associated with their pre- and post-migration experiences of loss, terror, and disruption (Bronstein & Montgomery, 2011; Fazel et al., 2012). Refugees aged 18 years or younger, resettled in western countries, have an 11% chance of developing posttraumatic stress disorder (PTSD; American Psychiatric Association, 2000; Fazel, Wheeler, & Danesh, 2005). Those under 25 have a 19–54% chance of developing PTSD, and a 3–30% chance of developing depression (Bronstein & Montgomery, 2011).

It has been suggested that adolescent refugees with social, behavioural, and mental health problems are reluctant to seek mental health care (De Anstiss, Zaian, Procter, Warland, & Baghurst, 2009). Moreover, the mental health needs of this group are often not recognised (e.g., Bean, Eurelings-Bontekoe, Mooijaart, & Spinhoven, 2006). Given the high prevalence of mental health problems among refugee youth, mental health service systems must rise to the challenge of providing culturally appropriate, accessible, and effective services (Ehntholt & Yule, 2006).

Although many researchers highlight the importance of the PTSD concept in understanding and treating young refugees (e.g., Hodes, 2000), this perspective has been criticised for “minimising the role of culture” (Bracken, Joan, & Summerfield, 1995), “oversimplifying experiences” (Richman, 1993), and “pathologising normal stress responses” (Kleber, 1995). A shift in focus has been to move away from the negative aspects related to being a refugee towards emphasising positive aspects and resilience in the face of adversity. A focus on resilience in young refugees may aid in adequately representing their response to adversity, understanding their needs, and shaping any interventions. The aim of this article is to describe achievements and challenges in the field of resilience in relation to young refugees and to address methodological issues and paths for future research. These form the background for the presentation of a study proposal into resilience of young refugees.

## Defining and measuring resilience: achievements and challenges

The field of resilience knows several achievements and challenges, in particular with reference to young refugees.

### Definition

The study of resilience originated in the 1970s with a group of researchers who directed their attention to the investigation of children capable of progressing through normal development despite exposure to significant adversity (Masten, 2001). Resilience is a concept that is intuitively understood (Brom & Kleber, 2009) but, in fact, variously defined. Generally, resilience refers to positive adaptation, or the ability to sustain or regain mental health, despite experiencing significant adversity (Wald, Taylor, Asmundson, Jang, & Stapleton, 2006). Although definitions evolved as scientific knowledge has grown, there is still little consensus. Discrepancy exists in the conceptualisation of resilience as a personal trait versus a dynamic process. If resilience is a personal trait, the absence of it might lead to stress-related psychopathology, and even in non-trauma-exposed individuals resilience can be measured. Masten (1994) warns against using the term “resiliency” in such a way because it paves the way for perceptions that some individuals simply do not “have what it takes” to overcome adversity. Besides, such a term does little to illuminate processes underlying resilience or to guide the design of appropriate interventions (e.g., Masten, Best, & Garmezy, 1990). Others (e.g., Luthar, Cicchetti, & Becker, 2000) see resilience as a dynamic process encompassing positive adaptation within a context of significant adversity. They propose that resilience-related characteristics develop in reaction to environmental challenges, and that these characteristics may only present themselves in response to trauma exposure.

In young refugees, especially, it appears inappropriate to consider mental health problems as proof of lack of resilience. Some argue that the psychological problems presented by young refugees should be considered as normal reactions to abnormal circumstances (Papadopoulos, 1999). Furthermore, it seems inappropriate to define resilience as the ability “to bounce back”. For young refugees a return to “normal” life is impossible. In this case, the metaphor by Walsh (2002) may be more appropriate: she describes resilience as “bouncing forward” in the face of an uncertain future.

### Assessment

While broad descriptions of resilience make it possible to encompass the complexities of this construct and provide a framework for understanding it (Brom & Kleber, 2009), different and complex definitions make it difficult to measure “resilience”. Challenges in measuring resilience vary from the issue of cross-cultural equivalence to the way one measures “exposure to significant threat or severe adversity” and the quality of “positive adaptation” among individuals at risk. Resilience may be evaluated as a decrease or an absence of psychopathology (Scales, Benson, Leffert, & Blyth, 2000), success in meeting developmental milestones (Masten & Coatsworth, 1998), or a high state of wellbeing. Besides, resilience is often linked to a specific area, such as “educational resilience”, “emotional resilience”, or “cultural resilience” (Clauss-Ehlers, 2004). Researchers must specify the particular areas to which their data apply and must clarify that success in these domains by no means implies positive adaptation across all functional areas (Luthar et al., 2000). In addition to these general concerns, a limitation of many studies about young refugees is their frequent reliance on the answers of informants such as parents rather than on information provided by the young refugees themselves. Young refugees’ own perceptions are further obscured by the interpretations of Western researchers and by using questionnaires designed for Western populations.

### Relation to other constructs

Resilience research has successfully drawn attention away from a problem-oriented approach to consequences of adversity to a broader view of human experience that includes an understanding of individual strengths and the ability to deal with shocking events. Other constructs addressing similar issues have been, for example, coping, benefit finding, and posttraumatic growth. How these constructs relate to resilience is still relatively unclear. Only a few studies examine the potential overlap or interaction among these constructs. Cambel-Sills, Cohan, and Stein (2006) show that task-oriented coping is positively and emotional-oriented coping is negatively related to resilience, and that resilience moderates the relationship between childhood emotional neglect and current psychiatric symptoms in young adults. Research on refugee youth found that poor emotion-focused coping is associated with lower quality of life (Huijts, Kleijn, Van Emmerik, Noordhof, & Smith, 2012). Posttraumatic growth focuses on the experience of positive transformation that occurs as a result of the struggle with trauma (Cryder, Kilmer, Tedeschi, & Calhoun, 2006), while resilience focuses on positive adjustment despite significant life adversity. Although growth and resilience are distinct constructs with distinct posttraumatic pathways, there may be some interplay between the two constructs (Meyerson, Grant, Carter, & Kilmer, 2011). No quantitative study has focused specifically on post-traumatic growth in young refugees (Pacione, Measham, & Rousseau, 2013), although posttraumatic growth has been identified in children and adolescents (Alisic, Van der Schoot, Van Ginkel, & Kleber, 2008; Clay, Knibbs, & Joseph, 2009; Meyerson et al., 2011) as well as in refugee and immigrant adults (Ai Tice, Whitsett, Ishisaka, & Chim, 2007; Berger & Weiss, 2006; Hussain, & Bhushan, 2013; Kroo & Nagy, 2011; Powell, Rosner, Butollo, Tedeschi, & Calhoun, 2003; Teodorescu et al., 2012). The only qualitative study of posttraumatic growth in refugee adolescents (Sutton, Robins, Senior, & Gordon, 2006) shows that the degree to which each of the participants had experienced growth varied, and that they experienced co-existence of on-going distress and positive changes.

### Biological factors

Recent research has shown that “resilience is mediated by adaptive changes in several neural circuits involving numerous neurotransmitter and molecular pathways” (for an overview see Feder, Nestler, & Charney, 2009, p. 446). A multi-informant twin study demonstrated that about one quarter of the total variation in trait resilience in adolescents is attributable to environmental factors, while additive genetic factors explained nearly three quarter of the variation (Waaktaar & Torgersen, 2012). In a refugee population, researchers found further evidence for the gene–environment interplay in PTSD (Kolassa et al., 2010). This study revealed that genetic influences lose importance when environmental factors cause an extremely high trauma burden to an individual. Nevertheless, the environmental, genetic, epigenetic, and neural mechanisms that underlie resilience have been under-examined. To date, to the best of our knowledge, there have been no empirical studies that investigate whether there are particular gene variants that may be related to resilience in young refugees.

### External factors

The literature on resilience has focused mostly on individual attributes such as personality traits and biology (Bonanno, 2004; Yehuda, Flory, Southwick, & Charney, 2006), with relatively little focus on the degree to which these attributes are themselves dependent on external factors such as supportive relationships. The absence of social support and the presence of contextual life stress have been shown to be important risk factors for the development of PTSD (Brewin, Andrews, & Valentine, 2000; Olff, 2012; Zepinic, Bogic, & Priebe, 2012). This has been the case for refugee children and adolescents as well (Thomas & Lau, 2002). Montgomery (2010) showed that the number of types of stressful events after arrival in the host country was correlated with persistence of psychological problems in young refugees. Whether the presence of social support or the absence of contextual life stress increases the likelihood of resilience in young refugees remains to be studied.

In conclusion, while the field of resilience research has increased, our understanding of resilience, current difficulties exist with respect to its: 1) definition; 2) assessment; 3) relatedness to other constructs; 4) underlying biological factors; and 5) external factors, and overall intercultural generalisability. Empirical evidence suggests that the determinants of resilience are complex, with social, psychological, and biological (genetic) factors all believed to contribute. Studies of posttraumatic functioning in young refugees have highlighted “weaknesses and deficits”, while “simultaneously overlook(ing) the strengths and resources that enable children to grow and thrive in the face of seemingly overwhelming challenges” (Berman, 2001, p. 248). Consequently, further research is needed to achieve a better understanding of the construct of resilience in young refugees.

## Paths for future research

Because of its multi-faceted character, future research into resilience cannot rely on a single method and may profit from a diversity of research approaches. Below, we describe four research strategies that are deemed useful for future resilience research in young refugees.

### Qualitative research and qualitative syntheses

Qualitative approaches focus on subjective feelings, meanings, and experiences and in doing so make it understandable as to why people behave in particular ways (Bowling, 2002). Britten (2011) argues that qualitative research can explain the how, why, and what is going on. It can account for cross-cultural diversity in individual contexts by producing authentic results that reflect the lives of the people studied (Ungar & Nichol, 2002). Therefore, qualitative methods seem especially suited to examine young refugees’ own perspective on the phenomenon of resilience.

Qualitative evidence synthesis is the interpretation and synthesis of all qualitative research that can be detected in a particular scientific area (Hannes & Lockwood, 2012). This relatively new method develops quickly and uses similar systematic steps as are used in systematic reviews of quantitative research. Qualitative evidence syntheses are frequently used in social sciences and health care in order to systematically review what is already known, what knowledge gaps can be detected, and to add to the evidence base underlying interventions (Dixon-Woods, Fitzpatrick, & Roberts, 2001). The field of mental health and adaptation to stressful experiences in young refugees can benefit from such a systematic overview of qualitative studies to build a cumulative evidence base and to guide and target new primary qualitative research, in particular to address cross-cultural differences (Jones & Kafetsios, 2002; Van Wesel, Boeije, Alisic, & Drost, 2012).

### Mixed methods

In order to get a clearer and broader picture of trauma topics, including the construct of resilience, a mixed methods approach appears promising, especially with underserved groups (Creswell & Zhang, 2009). Mixing different methods offers the opportunity to respect the strengths of different methods and to include them in the planning of a single research project to enhance the balance and authoritativeness of conclusions (Dattilio, Edwards, & Fishman, 2010). For example, while quantitative methods can be used to make more general conclusions and to verify qualitative hypotheses, qualitative research can give more in-depth stories and answers to how different factors are related to each other. Combined, they can achieve a more holistic understanding of refugee youth and eventually help guide therapeutic interventions. Undertaking mixed methods research requires a research team with a range of skills and expertise of the research process and recognition of the underlying complexities. The integration of the quantitative and qualitative component to produce the surplus value is a major challenge of mixed method research (Boeije, Slagt, & Van Wesel, 2013; O'Cathain, Murphy, & Nicholl, 2010). Guidelines are increasingly available for integrating and reporting mixed methods research (Curry et al., 2013; Pluye, Gagnon, Griffiths, & Johnson-Lafleur, 2009).

Creswell and Zhang (2009) demonstrated how the field of trauma research could profit from mixed methods designs. In the field of childhood trauma, a narrative review presents different objectives for the use of mixed methods designs: measures and meaning, intervention evaluation, theory building, and the development of measurement instruments (Boeije et al., 2013). Mixed methods can also add value in resilience research in young refugees, because they “can help to explain both local constructions of resilience that are relevant to culturally and contextually distinct settings and the generalizability of the protective processes that are identified” (Ungar, 2012, p. 387).

### Comparative studies

Comparing different populations can be a good strategy to identify factors associated with resilience. In particular, this may be achieved by comparing participants representing extreme ends of a spectrum (Yehuda et al., 2006). Within the current context, that would preferably involve subjects exposed to war and migration versus unexposed subjects. Furthermore, a cross-cultural comparison may help to identify local and global factors that explain resilience. Investigating the tension between aspects of resilience that are shared and those that are distinct to a specific cultural group contributes to a social–ecological understanding of resilience (Ungar, 2011).

### Longitudinal studies

Longitudinal studies, involving assessments at more than two points in time, are needed to see the range of characteristics or responses to adversity and how these change over time. Furthermore, they are needed to clarify the discussion about the definition of resilience, resilience as a personal trait versus a dynamic process, and the influences of external factors. Little is known about the longitudinal course of psychopathology in refugee youth (Almqvist & Brandell-Forsberg, 1997; Bean, Eurelings-Bontekoe, & Spinhoven, 2007; Becker, Weine, Vojvoda, & McGlashan, 1999; Hjern & Angel, 2000; Krupinski & Burrows, 1986; Montgomery, 2010; Rousseau, Drapeau, & Rahim, 2003; Sack, Him, & Dickason, 1999), let alone of the more complex process of resilience. Longitudinal studies are necessary to further understand the process of resilience, that is, when and how adaptation takes place (Montgomery, 2010).

## Studying young refugees’ resilience combining different methods

Above we have argued that the field of resilience in refugee youth will benefit from the use of different methods. Here, we describe a specific research project that incorporates these methods and that is in its early stages. The project focuses on resilience of young refugees and their Dutch peers and involves 1) qualitative, 2) quantitative, 3) experimental, and 4) psychobiological components. Given below is a description of the sequence and contents of each of these components.

1. The project has started with a qualitative evidence synthesis, that is, a meta-ethnography (Noblit & Hare, 1988) of qualitative studies on resilience in refugee youth. A total of 25 studies were identified and are currently being analysed and synthesized. The findings from the meta-ethnography will guide the primary interview study that we are going to conduct; contrasting, missing, or ambiguous information and third-order interpretations, derived from the meta-ethnography, will be taken on board by developing the interview study. We will interview refugee adolescents (until saturation has been reached) to obtain in-depth stories of their needs, experiences, and coping styles. This enables us to get a better understanding of their perspective of fleeing and living in the Netherlands.
2. Next, questionnaires will be used to examine the extent to which the degree of resilience is determined by background characteristics (for example, age, education, country of origin, religion, and length of residence in the Netherlands) and personal and social aspects, to determine how resilience is related to other constructs such as coping and posttraumatic growth, and to find out how resilience can be operationalized in terms of “health” and “wellbeing”. Questionnaires are partially based on the results of the qualitative studies; important or ambiguous topics that emerged during the interviews will be added, if possible, to the quantitative part by means of a questionnaire that is linked to this specific topic. This two-step approach to the survey adds to the validity of inquiry (Lee & Smith, 2012) as well as creates an awareness of protective processes that are culturally relevant or implicit. Adolescents (100 Dutch adolescents and 100 young refugees aged between 12 and 16 years) were asked to fill out questionnaires about personal and social factors, traumatic events, posttraumatic stress reactions, emotional and behavioural problems, quality of life, and posttraumatic growth (see Table 1 for the list of questionnaires). These questionnaires have been chosen for their good psychometric qualities and their suitability for youngsters as well as heterogeneous cultural populations. The questionnaires enable us to determine whether “resilient youth”, as measured by the Wagnild and Young resilience-scale (1993), report fewer symptoms and behavioural problems and score more positive on mental well-being than “less resilient” youth, and how social factors such as culture, acculturation, social support, refugee status, and discrimination influence (refugee) youth. Wagnild and Young's resilience scale appears to be the best tool to measure resilience in adolescents (Ahern, Kiehl, Lou Sole, & Byers, 2006), because of its adequate psychometric properties when compared to other questionnaires and its use in culturally heterogeneous populations.
3. An experimental setting (The Behavioral Indicator of Resiliency to Distress (BIRD); Lejuez, Daughters, Danielson, & Ruggiero, 2006) will be used to see how these adolescents deal with frustration, and whether “resilient” adolescents have a higher distress tolerance than “less resilient” youngsters. The BIRD measures distress tolerance by determining how long a participant persists on a computer task that becomes increasingly difficult.
4. Finally, we will investigate genetic predictors of resilience and tolerance to distress, looking at the variants of the serotonin transporter gene (5-HTTLPR) and the SLC6A3 dopamine transporter (DAT). Amstadter et al. (2012) suggest that distress tolerance (also measured with the BIRD) is at least partially regulated by specific genetic variants, like the 5-HTTLPR polymorphism. Stein, Campbell, and Gelernter (2009) report that the long version of this gene is associated with emotional resilience in students; De Neve (2011) conclude that adolescents with two long variants of this gene appear to be happier than adolescents with two short variants of this gene.

**Table 1:** Questionnaires that will be used in the proposed research

	Questionnaire	Authors
Personal factors		
Self-esteem	The Rosenberg Self Esteem Scale	Rosenberg (1965)
Optimism	Life Orientation Test	Scheier & Carver (1985)
Personality	The Big Five Inventory	John, Donahue & Kentle (1991)
Coping	Adolescent Coping Orientation for Problem Experiences	Patterson & McCubbin (1987)
Social factors		
Social support	Multidimensional Scale of Perceived Social Support	Zimet, Dahlem Zimet & Farley (1988)
Discrimination	Everyday Discrimination Scale (partial)	Williams, Yan Yu & Anderson (1997)
Acculturation	The Lowlands Acculturation Scale (partial)	Mooren, Knipscheer, Kamperman, Kleber & Komproe (2001)
Posttraumatic stress	Children's Revised Impact of Event Scale	Children and War Foundations (1998)
Emotional and behavioural problems	The Strengths and Difficulties Questionnaire	Goodman, Meltzer & Bailey (1998)
Quality of life	The Satisfaction with Life Scale	Diener, Emmons, Larsen & Griffin (1985)
Resilience	Resilience Scale	Wagnild & Young (1993)
Posttraumatic growth	Posttraumatic Growth Inventory for Children	Kilmer et al. (2006)

In summary, this proposed research incorporates different culture-sensitive sources of information by different populations (young refugees and their Dutch peers) and looks at the interrelationship between individuals and contexts.

## Conclusion

Building on the current body of research, there remains much to discover about the processes underlying the development of resilience in refugee youth. Our current research project aims to contribute to existing knowledge by incorporating a multi-method design within a social ecological framework. Qualitative (meta-ethnography, interview study), quantitative (survey), experimental (BIRD computer-task), and psycho-biological (serotonin transporter gene 5-HTTLPR and the SLC6A3 dopamine transporter) information is gathered in a group of young refugees and their Dutch peers. Working as a multidisciplinary team, we trust to be able to combine, compare, and integrate distinct perspectives on the ability to adjust. In this way, we hope to achieve a better understanding of the construct of resilience and protective processes that are culturally relevant. Moreover, we have set out to come up with recommendations for ways to improve resilience in these youngsters.

This proposed study cannot solve all of the challenges in the field of resilience, and caution should be exercised when interpreting the results cross-culturally. If resilience is a dynamic developmental process, the scope of a cross-sectional design study such as this is limited and a longitudinal research is needed. In addition, most assessments are person-based; taking little account of the social context of the participants. This study uses a common way to mix different methods in a research project: it analyses qualitative and quantitative data separately. However, it is preferable to integrate different methods throughout the analysis phase of a research project which can lead to a richer integration across methods and analyses (Yoshikawa, Weisner, Kalil, & Way, 2008). Nevertheless, integrating different methods is still an unconcerned area with a lot of methodological difficulties.

The concept and study of resilience offers important opportunities for empowerment of traumatised groups. While the legal label of being a refugee is life-saving, the social label carries with it a risk of prejudice and stigma (Zetter, 1991). The stereotypical perception of young refugees as adolescents at risk is not without consequences: it is difficult to spread one's wings when pigeonholed. It is of great social importance to see refugee youth not as passive victims without capacities but as survivors with social potential who can inspire with their ability to “bounce forward”.
